# Relevance of reactive oxygen species in liver disease observed in transgenic mice expressing the hepatitis B virus X protein

**DOI:** 10.1186/s42826-020-00037-1

**Published:** 2020-02-26

**Authors:** Dae-Yeul Yu

**Affiliations:** Korea Research Institute of Bioscience and Biotechnology (KRIBB), 125 Gwahak-ro, Yuseong-gu, Daejeon, 305-806 South Korea

**Keywords:** Hepatitis B virus (HBV), Hepatitis B virus X (HBx), Hepatocellular carcinoma (HCC), Hepatic steatosis, Hepatic fibrosis, Hepatic glucose, Reactive oxygen species (ROS), Oxidative stress, Transgenic mice

## Abstract

The hepatitis B virus (HBV) infects approximately 240 million people worldwide, causing chronic liver disease (CLD) and liver cancer. Although numerous studies have been performed to date, unfortunately there is no conclusive drug or treatment for HBV induced liver disease. The hepatitis B virus X (HBx) is considered a key player in inducing CLD and hepatocellular carcinoma (HCC). We generated transgenic (Tg) mice expressing HBx protein, inducing HCC at the age of 11–18 months. The incidence of histological phenotype, including liver tumor, differed depending on the genetic background of HBx Tg mice. Fatty change and tumor generation were observed much earlier in livers of HBx Tg hybrid (C57BL/6 and CBA) (HBx-Tg hybrid) mice than in HBx Tg C57BL/6 (HBx-Tg B6) mice. Inflammation was also enhanced in the HBx-Tg B6 mice as compared to HBx-Tg hybrid mice. HBx may be involved in inducing and promoting hepatic steatosis, glycemia, hepatic fibrosis, and liver cancer. Reactive oxygen species (ROS) generation was remarkably increased in livers of HBx Tg young mice compared to young wild type control mice. Previous studies on HBx Tg mice indicate that the HBx-induced ROS plays a role in inducing and promoting CLD and HCC.

## Introduction

The hepatitis B virus (HBV) infection is prevalent worldwide, causing liver disease and liver cancer. HBV, a member of the *Hepadnaviridae* family, is a small DNA virus similar to retrovirus [1]. The viral genome encodes four known genes: C, X, P, and S. The core protein is coded for by gene C (HBcAg and HBeAg), depending on whether translation initiates from the core or pre-core regions, respectively [2]. The *S* gene encodes the viral surface envelope proteins (HBsAg), and can be structurally and functionally divided into the pre-S1, pre-S2, and S regions. The P gene encodes the large polymerase protein (pol; about 800 amino acids). The HBV X (HBx) gene encodes a 16.5-kd protein (HBxAg) having multiple functions, including signal transduction, transcriptional activation, DNA repair, cell cycle control, and inhibition of protein degradation [3–6].

Persistently high levels of HBV replication are suggested to be correlated with the progression of chronic liver disease (CLD) to hepatocellular carcinoma (HCC) [7]. A correlation between virus replication and CLD may, therefore, contribute to determining disease progression. Briefly, fragments of HBV DNA, mostly encoding the HBx, become integrated at multiple sites within the host DNA [8]. These integration events result in enhancing the intrahepatic expression levels of HBx that alter patterns of host gene expression. Although the relatively low levels of HBx derived from the virus mini-chromosome support expression and replication of the virus gene, it was hypothesized that increase of intrahepatic levels of HBx epigenetically alters the expression patterns of selected host genes, contributing to both virus persistence and malignant transformation [9, 10]. Recently it is reported that nuclear HBx binds the HBV minichromosome and modifies the epigenetic regulation of the covalently closed circular DNA (cccDNA) function [11] and HBx protein contributes to liver carcinogenesis by H3K4me3 modification through stabilizing WD repeat domain 5 protein [12]. Therefore, inhibition of HBV transcription from cccDNA is indicated to be a therapeutic target towarding a functional HBV cure.

In patients infected with HBV, HBx often co-exists with HBe in serum [13] and as replication complexes (with HBcAg) in the liver [14]. HBxAg was present most often in sera positive for HBeAg and/or HBV DNA or apparently complexed to anti-HBx in sera lacking these markers [13]. HBx is required to initiate and maintain HBV replication in HepaRG cells [15] and human-liver-chimeric mice [16]. Thus, HBx expression is associated with virus replication. In connection with the fact that HBx plays a key role in HBV replication, the expression and activity of HBx is stimulated in an oxidative environment. An oxidative environment could be created in HBV infected cells by virtue of the association of HBx with mitochondria [17]**.** Oxidative stress induced by HBx in the endoplasmic reticulum activates the unfolded protein response and expression of pro-inflammatory cyclooxygenase-2 through the activation of transcription factor 4 pathway [18]. In addition, oxidative stress induced by mitochondrial associated HBx activates selected transcription factors, such as NF-ĸB, STAT3 and activating protein 1 [19].

Reactive oxygen species (ROS) are derived from the metabolism of molecular oxygen [20], and include the superoxide anion radical (O_2_^-.^), singlet oxygen (^1^O_2_), hydrogen peroxide (H_2_O_2_), and the highly reactive hydroxyl radical (^.^OH). The harmful effects of oxygen emanate from its metabolic reduction to these highly reactive and toxic species [19]. ROS normally exist in all aerobic cells balanced with biochemical antioxidants. Oxidative stress occurs when this critical balance is disrupted due to excess ROS, antioxidant depletion, or both. Given that ROS production is a natural process, and that persistently high levels of ROS could be damaging, the human body has developed antioxidant systems for their neutralization. Various enzymatic and non-enzymatic mechanisms have evolved to protect cells against ROS. Enzymatic antioxidants include superoxide dismutase (SOD, which detoxifies the superoxide ion), and catalase, glutathione peroxidase (GPx) and peroxiredoxins (Prxs), which inactivate H_2_O_2_. All Prx enzymes contain a conserved cysteine (Cys) residue that undergoes a cycle of peroxide-dependent oxidation and thiol-dependent reduction during catalysis. There are 6 isoforms of Prx (Prx I to VI) in mammalian cells, which are classified into 3 subgroups (2-Cys, atypical 2-Cys, and 1-Cys) based on the number and position of Cys residues that participate in catalysis [21]. Several researches have reported that Prxs are associated with CLD [22–24].

A correlation between HBx staining and the intensity of CLD is expected in this chronic pro-inflammatory environment. Relatively low levels of intrahepatic HBx staining were found in biopsy samples from patients with low grade hepatitis, whereas intense and widespread HBx staining has been observed in biopsies from advanced fibrosis and cirrhosis [9, 25], indicating a direct correlation between HBx staining and liver damage. The relationship of HBx expression to disease severity is also consistent with the idea that when the liver regenerates, fragments of HBV DNA encoding the HBx region increasingly integrate into multiple regions of the host genome during normal host DNA replication, resulting in increasing accumulation of intrahepatic HBx with CLD progression [26]. Thus, persistent inflammation in a chronically damaged liver may result in the development of HCC, despite low levels or undetectable levels of virus replication [26].

## Main text

### Generation of HBx Tg mice inducing liver cancer

Until the 1990’s, chronic infection with HBV was regarded as one of the major causes of HCC. However, there was no evidence to prove that the HBV gene is involved in tumor development. In 1987, through identification of a viral regulatory gene HBx, the virus was suggested to be directly involved in HCC. The HBx protein alters the host gene expression and results in the development of HCC by acting as a transcriptional transactivator of viral genes [27]. Therefore, transgenic (Tg) mice expressing HBx were generated in several laboratories to prove whether HBx may play a key role in inducing HCC. Kim et al. [28] reported that HBx induces liver cancer in Tg mice. A high incidence of HCC was found in Tg mice, subsequent to microinjecting a 1.15 kb HBV subtype *adr* DNA fragment with nucleotide positions 707 to 1856 in the viral genome. In contrast, the HBx Tg mice generated in other laboratories developed no obvious hepatic pathology, although they expressed the X-gene in liver cells and the HBx protein could be detected in some cases [29–32]. Therefore, HBx was concerened with an oncogenic role. We demonstrated to generate HBx Tg mice to confirm whether HBx induces liver cancer [33]. The X-gene construct with an HBV-X gene under authentic promoter was microinjected into hybrid (C57BL/6 x DBA) eggs. We consistently observed grossly defined HCC in mice expressing the X-protein through 6 generations, from the ages of 11 to 18 months. The incidence of HCC (86%) in our HBx Tg mice may be highly significant, since the HBV X-gene Tg mice produced in other laboratories did not develop liver tumor or any other pathologic phenomena, except for one case [28]. Since then, Wu et al. have generated HBx Tg mice to investigate in vivo effects of HBx on hepatocyte proliferation and viability during liver regeneration of the HBx Tg mice post-hepatectomy [34]. They injected the albumin HBx construct into embryos of C57BL/6 mice, and succeeded in generating HBx Tg mice that developed HCC at around 14–16 months of age. Wang et al. also introduced the HBx gene into the p21 locus of the mouse genome by homologous recombination, to better understand the consequences of integrating the HBV gene [35]. The HBx gene knock-in Tg mice expressed HBx mainly in the liver tissues, and developed HCC.

As mentioned above, HBx Tg mice have been generated in eight laboratories till date (Table 1). Of these, four laboratories succeeded in generating HBx Tg mice inducing HCC, which did not develop in the other 4 laboratories. Although HCC was caused in all bi-transgenic animals derived by crossing HBx Tg mice with WHV/c-myc oncomice in which liver specific expression of c-myc was driven by woodchuck hepatitis virus (WHV) regulatory sequences [36].

**Table 1:** HBx transgenic mice published

HBV DNA subtype	Promoter	Mouse strain	Tumor generation	Published paper
adr	HBV X	CD-1	HCC	Kim et al. (Nature 1991) [28]
adr	HBV X	F1 hybrid (C57BL/6 X DBA)	HCC	Yu et al. (J of Hepatology 1999) [33]
ayw	SV40, ATIII, HBV X	F1 hybrid (C57BL/6 X DBA)	No serious liver damage	BILLET et al. (J of Virology 1995) [29]
adw	Alpha1 antitrypsin	ICR females X B6C3F1 males.	No tumor	Lee et al. (J of Virology 1990)
ayw	HBV X	F1 hybrid (C57BL/6 X DBA)	No description	Perfumo et al. (J of Virology 1992) [31]
No description	HBV X, Rat insulin II	C57BL/6, F2 hybrid(C57BL/6 X DBA)	No pathological change	Reifenberg et al. (J of Hepatology 1997) [32]
ayw	albumin	C57BL/6	HCC	Wu et al. (BBRC 2006) [34]
No description	HBV X in p21 locus	C57BL/6	HCC	Wang et al. (Hepatology 2004) [35]

It is difficult to understand why four laboratories failed to observe HCC in their HBx Tg mice. Although certain factors, mouse strain, HBV genotype and HBx integration site were considered to be responsible, it is difficult to deduce which factor is involved in achieving success or failure of inducing HCC in HBx Tg mice. This is because the same mouse strain and HBV genotype were cross-used in generating HBx tg mice, and HBx integration site was not identified in all HBx Tg mice. However, considering all results, it is apparent that HBx has an oncogenic role in hepatic tumorigensis in Tg mice.

### Difference between hepatic phenotypes, depending on the HBx Tg mouse strains

As previously mentioned [33], HBx Tg mouse was generated using the F1 hybrid mouse strain (C57BL/6 X DBA), since hybrid embryos are harder than inbred embryos and microinjection of HBx transgene into embryos is easier. After confirming that HBx Tg mice induced HCC, we generated HBx homozygous Tg mice (HBx^+/+^Tg) using the strains C57BL/6, hybrid (C57BL/6 X CBA), and CBA, to understand the phenotypes depending on their genetic background. Compared to wild type (WT) mice, the body weight significantly increased from about 6 weeks of age to old age in the HBx Tg mice regardless of strain, suggesting that HBx expression in hepatocytes induces increase in body weight as compared to WT. HBx Tg hybrid (C57BL/6 X CBA) (HBx-Tg hybrid) mice show about 2–3-fold increased body weight than the HBx Tg C57BL/6 (HBx-Tg B6) mice. In addition, HBx-Tg hybrid mice were larger in size than HBx-Tg B6 mice. However, a sufficient number of HBx-Tg CBA mice were not obtained to examine the phenotype due to difficulty of breeding the mice, resulting in no data on HBx-Tg CBA mice. We could only determine that the size of HBx-Tg CBA mice is similar to HBx-Tg hybrid mice.

Assessment of the histological phenotype revealed features associated with hepatic tumorigenesis in liver of WT and HBx Tg mice (Tables 2, 3). Fatty change appeared in HBx Tg mouse livers, but not in WT mouse livers. Especially, micro-fatty changes were observed in 31.9% of HBx-Tg hybrid mice at the age of ~ 12 weeks, but were absent in WT mice at the same age. Moreover, micro-fatty changes appeared in 6.7% of HBx-Tg B6 mice, but not in WT mice at the same age. However, macro-fatty changes were observed in 4.3% of HBx-Tg hybrid mice, but not in WT and HBx-Tg B6 mice at the same age. Fatty change was observed in all HBx-Tg B6 mice at the age of 28~44 weeks, and in all HBx-Tg hybrid mice at the age of 28~36 weeks. These data suggest that the greater body weight of HBx-Tg hybrid mice than HBx-Tg B6 mice may be associated with the increased fatty changes observed in HBx-Tg hybrid livers in young mice. Inflammation was observed in the livers of 9.8~50% HBx Tg mice examined, and was observed to be augmented in HBx-Tg B6 mice as compared to HBx-Tg hybrid mice. Inflammation was detected in 22.2% HBx-Tg B6 mice and in 17% HBx-Tg hybrid mice at the age of ~ 12 weeks, but not in WT mice at the same age. These data also suggest that HBx induces inflammation in the livers of HBx Tg mice. However, the rate differs in accordance with the strain and age of HBx Tg mice. Necrosis was almost absent in the livers of HBx-Tg hybrid mice, but minimally detected in the livers of HBx-Tg B6 mice. Regardless of the strain, dysplasia was observed in almost all HBx Tg mice from a young age, suggesting that HBx is involved in abnormal growth of hepatocytes. Dysplastic nodules were found at 12~28 weeks age in HBx-Tg hybrid mice; however, it was remarkably observed only in later stages of HBx-Tg B6 mice (44~64 weeks age). Hepatic tumors were detected in 87.5% HBx-Tg hybrid mice at the age of 28~36 weeks, but were observed in 62.5% HBx-Tg B6 mice at the advanced age of 44~64 weeks. These results indicate that hepatic tumors develop earlier in the HBx-Tg hybrids than in HBx-Tg B6 mice.

**Table 2:** The histological phenotype in the liver of wild and HBx-Tg B6 mice

	Group 1			Group 2		
	WT	HBx Tg	*P*-value	WT	HBx Tg	*P*-value
Micro-fatty change	0/39(0%)	3/45(6.7%)	0.101	9/47(19.1%)	40/51(78.4%)	0.000
Macro-fatty change	0/39(0%)	0/45(0%)	–	2/47(4.3%)	25/51(49.0%)	0.045
Inflammation	0/39(0%)	10/45(22.2%)	0.002	9/47(19.1%)	5/51(9.8%)	0.187
Necrosis	0/39(0%)	1/45(2.2%)	0.349	0/47(0%)	4/51(7.8%)	0.050
Dysplasia	0/39(0%)	43/45(95.6%)	0.000	0/47(0%)	48/51(94.1%)	0.000
Dysplastic nodule	0/39(0%)	0/45(0%)	–	0/47(0%)	1/51(2.0%)	0.335
Tumor	0/39(0%)	0/45(0%)	–	0/47(0%)	0/51(0%)	–
	Group 3			Group 4		
Micro-fatty change	4/13(30.8%)	15/15(100%)	0.000	1/3(33.3%)	7/8(87.5%)	0.072
Macro-fatty change	0/13(0%)	15/15(100%)	0.000	0/3(0%)	3/8(37.5%)	0.214
Inflammation	1/13(7.7%)	3/15(20.0%)	0.353	1/3(33.3%)	4/8(50%)	0.621
Necrosis	1/13(7.7%)	1/15(6.7%)	0.916	0/3(0%)	2/8(25.0%)	0.338
Dysplasia	0/13(0%)	15/15(100%)	0.000	0/3(0%)	8/8(100%)	0.001
Dysplastic nodule	0/13(0%)	1/15(6.7%)	0.343	0/3(0%)	5/8(62.5%)	0.064
Tumor	0/13(0%)	1/15(6.7%)	0.343	0/3(0%)	5/8(62.5%)	0.064

Group 1: ~ 12 weeks, Group 2: 12~28 weeks

Group 3: 28~44 weeks, Group 4: 44~64 weeks

**Table 3:** The histological phenotype in the liver of wild and HBx-Tg hybrid mice

	Group 1			Group 2			Group 3		
	WT	HBx Tg	*P*-value	WT	HBx Tg	*P*-value	WT	HBx Tg	*P*-value
Micro-fatty change	0/34(0%)	15/47 (31.9%)	0.001	1/6 (16.7%)	12/14 (85.7%)	0.003	5/9 (55.6%)	8/8 (100%)	0.031
Macro-fatty change	0/34(0%)	2/47 (4.3%)	0.223	0/6 (0%)	4/14 (28.6%)	0.143	0/9 (0%)	8/8 (100%)	0.000
Inflammation	0/34(0%)	8/47 (17.0%)	0.011	0/6 (0%)	1/14 (7.1%)	0.502	0/9 (0%)	1/8 (12.5%)	0.274
Necrosis	0/34(0%)	1/47 (2.2%)	0.392	0/6 (0%)	0/14 (0%)	–	0/9 (0%)	0/8 (0%)	–
Dysplasia	0/34(0%)	46/47 (97.9%)	0.000	0/6 (0%)	13/14 (92.9%)	0.000	0/9 (0%)	8/8 (100%)	0.001
Dysplastic nodule	0/34(0%)	0/47 (0%)	–	0/6 (0%)	4/14 (28.6%)	0.143	0/9 (0%)	7/8 (87.5%)	0.000
Tumor	0/34(0%)	0/47 (0%)	–	0/6 (0%)	2/1414.3%)	0.329	0/9 (0%)	7/8 (87.5%)	0.000

Group 1: ~ 12 weeks, Group 2: 12~28 weeks, Group 3: 28~36 weeks

Taken together, the results indicate that fatty changes and tumor generation are observed much earlier in livers of the HBx-Tg hybrid mice than in HBx-Tg B6 mice. Furthermore, the histological phenotypes associated with hepatic tumorigenesis are different, depending on the strain of HBx Tg mice, suggesting that genetic backgrounds affect phenotypes of HBx Tg mice.

### HBx induces hepatic steatosis in Tg mice

It was suggested that chronic HBV infection is frequently associated with hepatic steatosis, which is a common histologic feature of chronic infection with hepatitis C virus (HCV) [37]. The frequency of hepatic steatosis ranges from 27 to 51% in subjects with a chronic HBV infection, and 31 to 72% in chronic HCV patients. Despite the high frequency of HBV-associated hepatic steatosis, the molecular mechanism of HBV or its viral proteins resulting in the pathogenesis of HBV-associated hepatic steatosis is unknown. We therefore investigated whether HBx may be involved in hepatic steatosis in HBx-Tg hybrid mice. As mentioned above, the body weight of HBx Tg mice significantly increases from about 6 weeks age to old age regardless of strain, when compared to WT mice, suggesting that HBx expression in hepatocytes induces increased growth of body weight. In addition, the liver weight per body weight of HBx-Tg hybrid mice increased 2-fold as compared with the WT mice at age 11-weeks. Moreover, H&E staining revealed the presence of diffused intracellular lipid droplets in liver sections of HBx-Tg hybrid mice. Increased lipid accumulation in livers of HBx-Tg hybrid mice was also observed by Oil-Red O staining. In addition, HBx-Tg hybrid mice show dysplasia as well as the fatty changes (Tables 2, 3). These results obtained in HBx-Tg hybrid mice, as well as lipid accumulation in HepG2 cells overexpressing HBx, indicate that HBx plays a role in the development of hepatic steatosis [38]. Hepatic fatty change was also observed in HBx Tg mice generated at other laboratories [34]. The molecular mechanism by which HBx causes lipid accumulation was therefore investigated and is summarized as follows. HBx leads to increased AKT activity and inhibition of PTEN expression, resulting in activation of sterol regulatory element binding protein 1(SREBP1), which upregulates hepatic lipid synthesis. The enhancement of CEBPα expression and activity by HBx results in increased PPARγ activity, thereby inducing the expression of hepatic adipogenic genes [38]. The molecular mechanisms by which HBx increases SREBP1 expression and transactivation were elucidated [39]. HBx interacts with liver X receptor α (LXRα) and enhances the binding of LXRα to LXR-response element (LXRE), thereby resulting in the upregulation of SREBP1 and fatty acid synthesis (FAS) to support lipogenesis in hepatic cells and HBx-Tg hybrid mice. In addition, increased ROS production further contributes to increased lipid droplet formation in HBx expressing cells [40]. Wu et al. also reported that liver fatty acid binding protein (FABP1) is dramatically expressed in the sera of HBV-infected patients and in both sera and liver tissues of HBV Tg mice, and may play a role in HBx-induced hepatic lipid accumulation via regulation of HNF3β, C/EBPα, and PPARα in the development of HBV-induced nonalcoholic fatty liver disease [41]. However, PPARα was not expressed in the HBx-Tg hybrid mice developed by us [38]. It is therefore necessary to confirm, in future studies, whether HNF3β and C/EBPα are involved in HBx induced hepatic lipid accumulation.

### HBx regulates hepatic glucose homeostasis in Tg mice

HBV infection is strongly associated with type 2 diabetes [42, 43], and diabetic hyperglycemia is associated with serum alanine aminotransferase activity in patients with HBV infection [44]. Chronic infection of HCV also develops a pathological feature of steatohepatitis. Inflammatory mediators, such as nitric oxide and TNF-α, have been shown to impair the metabolic action of insulin in the liver, which ultimately results in hepatic dysfunction causing insulin resistance. In fact, expression of inducible nitric oxide synthase (iNOS) is upregulated in the liver of chronic HBV-infected patients [45]. During acute or chronic HBV infection, HBx plays a key role in the induction of hepatic inflammatory responses, as proved by the fact that HBx transactivates the promoter of the iNOS gene through the proximal NF-κB binding site [46]. In addition, the HBV-induced TNF-α synthesis is transcriptionally up-regulated by HBx in hepatocytes [47]. However, the mechanisms through which HBV leads to glucose metabolism in chronic liver diseases were not elucidated. We therefore investigated the role of HBx in regulating glucose metabolism in HBx-Tg B6 mice and HBx-Tg B6 mice lacking iNOS. Livers of HBx-Tg B6 mice revealed increased expressions of gluconeogenic genes, similar to the HepG2 cells expressing HBx and non-tumor liver tissues of HBV patients with high expressions of HBx. This resulted in hyperglycemia due to increased glucose production. Importantly, the actions of HBx on hepatic glucose metabolism were thought to be mediated via iNOS signaling, as evidenced by the restoration of HBx-induced hyperglycemia, by suppressing the gene expression of gluconeogenic enzymes in iNOS knockout mice. Nitric oxide exposure of HepG2 cells expressing HBx caused a significant increase in the expression of gluconeogenic genes. Furthermore, hyperactivation of JNK1 in the liver of HBx-Tg B6 mice was also suppressed in the absence of iNOS, indicating the critical role of JNK in the mutual regulation of HBx- and iNOS-mediated glucose metabolism. These findings established a novel mechanism of HBx-driven hepatic metabolic disorder modulated by iNOS-mediated activation of JNK [48]. It was also noted that the overexpression of HBx proteins enhances the promoter activity of PEPCK and G6Pase in HepG2 cells [49]. Furthermore, although insulin attenuates the expression of the PEPCK and G6Pase genes, HBx inhibited the insulin-induced suppression of the promoter activity of these genes. Moreover, HBx proteins augment the SOCS3 expression and decrease IRS1 proteins, thereby causing impairments of hepatic insulin signaling and inhibition of hepatic insulin action. Taken together, these results suggest that HBx impairs hepatic insulin signaling by decreasing IRS1 proteins and increasing SOCS3 expression, indicating that HBx regulates hepatic glucose homeostasis by suppressing insulin signaling.

### HBx enhances the development of liver fibrosis in Tg mice

Liver fibrosis results from chronic damage to the liver, in conjunction with accumulation of extracellular matrix (ECM) proteins, including collagen [50]. Accumulation of ECM proteins induces fibrous scar formation, resulting in hepatic architecture distortion and subsequent regeneration of hepatocyte cirrhosis nodules. Moreover, cirrhosis brings about hepatocellular dysfunction and eventually progresses to liver cancer [51]. Chronic HBV infection induces liver fibrosis [52]. There are several convincing evidences that HBx correlates with the development of fibrosis. HBx induces severe lipid accumulation in HBx-Tg hybrid mice [38], and alters the production of ECM by modulating the expression of membrane-type matrix metalloproteinase-1 and fibronectin [53, 54]. More convincing evidence is the induction of liver fibrosis in HBV and HBx Tg mice exposed to carbon tetrachloride (CCl4) [55–57]. Gong et al. [57] reported that SATB1 expression is significantly up-regulated in liver fibrotic tissues from HBV chronic patients and HBV Tg mouse model, and knockdown of SATB1 in the liver significantly alleviates the fibrosis induced by CCl4 exposure in the HBV-Tg mouse model. Increased expression of SATB1 in hepatocytes enhances the activation and proliferation of hepatic stellate cells (HSCs) by secreting connective tissue growth factor (CTGF), Interleukin-6 (IL-6) and platelet derived growth factor-A (PDGF-AA). Their findings demonstrate that hepatic SATB1 upregulated by HBx exerts pro-fibrotic effects by paracrine activation of stellate cells in HBV-related fibrosis. Feng et al. [56] also investigated the significance of HBx in liver fibrosis during the progression of HBV-associated liver diseases, by assessing the upregulated P4HA2 in a model of fibrotic liver tissues from HBV-Tg mice, and clinical samples of liver fibrosis and liver cirrhosis. Interestingly, they found that HBx accelerates the collagen deposition in liver fibrosis through modulation of miR-30e targeting P4HA2 mRNA. The results provide novel perspectives on how HBx promotes liver fibrosis during development of HBV-associated liver diseases. In addition, Ahodantin et al. [55] examined whether HBx promotes liver fibrosis in full length (FL)-HBx Tg mice treated with CCl4, and observed that FL-HBx expression alters hepatocyte proliferation and potentiates CCl4-induced liver fibrosis, with increasing expression of inflammatory cytokines (TNF-α, TGF-β) and proteins (Collagen1a, α-Sma, PdgfR-β, MMP-13) involved in the epithelial mesenchymal transition (EMT), and severity of liver disease. These data suggest that HBx may be involved in liver fibrosis via different molecular signaling pathways each other in CCl4 treated HBx and HBV Tg mice.

### HBx induced oxidative stress may be involved in regulation of liver diseases, hepatic steatosis and liver cancer

Oxidative stress, an imbalance between the formation of ROS and antioxidant defense mechanisms, is well-documented to play a key role in pathological courses. Numerous studies using HBV-expressing cells as well as HBV-expressing mice, have verified that HBV infection induces oxidative stress. Oxidative stress and oxidative damage are also increased in patients with HBV infection [58–60]. Oxidative stress produce ROS that damages cellular molecules like lipids, proteins and DNA during chronic infection of HBV, ultimately resulting in the development of liver disease. Increased oxidative components or decreased antioxidants have been reported in acute or chronic HBV infections [61–64]. One study based on different cell models reports that HBV replication induces cellular ROS expression, thereby suggesting that HBV replication induces oxidative stress [65]. Of the proteins encoded by the HBV genome, HBx is a viral protein that plays a key role in oxidative stress, and is associated with the functioning of mitochondria, which is a major source of ROS. HBx, which are colocalized to mitochondria with a human voltage–dependent anion channel (HVDAC3), alters its transmembrane potential [66]. HBx also down-regulates mitochondrial enzymes related to the electron transport in oxidative phosphorylation (complexes I, III, IV, and V), sensitizes the mitochondrial membrane potential in a hepatoma cell line, and increases mitochondrial ROS and lipid peroxide production [67]. In addition, mitochondrially associated HBx activates transcription factors STAT-3 and NF-κB, and stimulates the mitochondrial translocation of Raf-1 via oxidative stress, which is relevant to liver disease pathogenesis associated with hepatitis B viral infection [17, 68]. HBx expression is also related with increased oxidative damage. It is reported that the C-terminal region of HBx is essential for mitochondrial DNA damage [69]. Consistently, Ren et al. measured the AP sites and γH2AX foci (a marker for DNA double strand breaks (DSB)) and reported that HBx plays a key role in HBV-induced oxidative stress, and HBx expression causes the oxidatively induced DNA damage associated with ROS [65]. These data indicate that HBx expression is associated with oxidative stress and DNA damage.

**Fig. 1:**
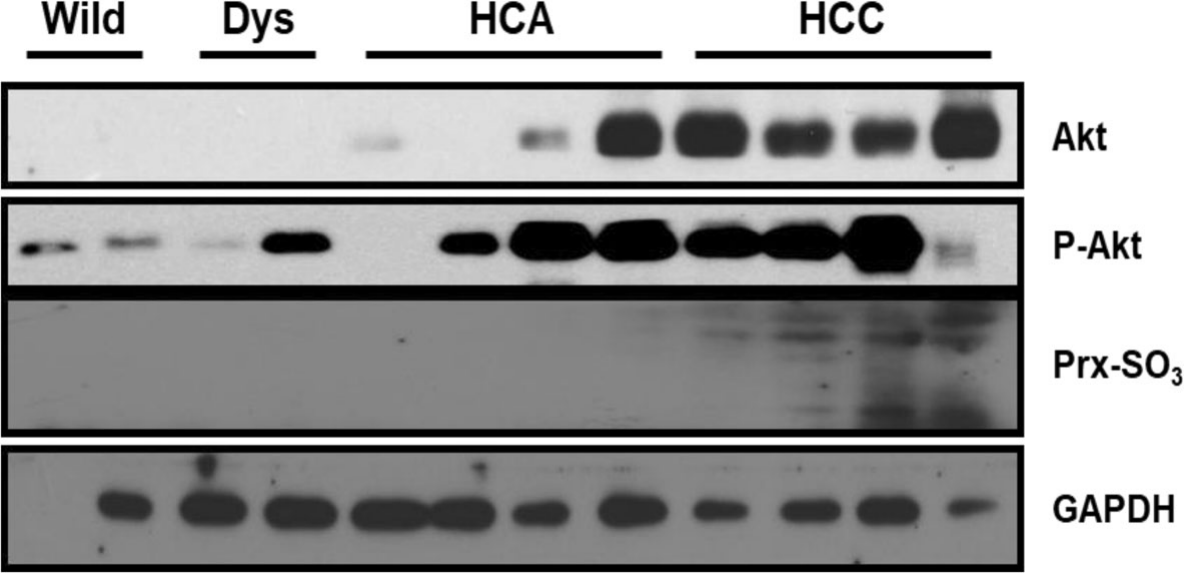
Western blot result showing expression levels of Akt, p-Akt, and Prx-SO_3_ in livers of HBx Tg mice. Glyceraldehyde 3-phosphate dehydrogenase (GAPDH) was used as a loading control. Wild; wild type, Dys; dysplasia, HCA; hepatocellular adenoma, HCC; hepatocellular carcinoma

As described above, mitochondrial dysfunction is a crucial pathophysiological factor in HBx-expressing hepatoma cells [67]. This suggests that HBx-induced mitochondrial dysfunction could be involved in liver diseases. It has been reported that patients with non-alcoholic steatohepatitis (NASH) present ultrastructural mitochondrial alterations [70] and increased ROS production [71, 72]. Therefore, mitochondrial dysfunction may play a key role in steatosis and steatohepatitis, by impairing fatty liver homeostasis and inducing the overproduction of ROS, that in turn trigger lipid peroxidation, cytokines release and cell death [73]. In association with these, accumulation of fatty change and dysplasia induced in our young HBx Tg mice suggest that mitochondrial dysfunction induced by HBx expression increases the ROS generation, which may cause steatosis and inflammation in HBx Tg mice. On maturing, liver cancer is induced in the HBx Tg mice by continuous accumulation of severe fatty changes and dysplasia (Tables 2, 3). As reported, ROS generation is remarkably increased in livers of HBx Tg mice at 4 weeks of age, compared with that of WT control mice [74]. The HBx-induced ROS promotes the Akt pathways via oxidized inactive PTEN in HBx Tg mice and HepG2-HBx cells [75]. In addition, we investigated in which stage of tumorigenesis Prx-SO_3_, a hyperoxidized form of Prx2 and Prx3, may increase in HBx Tg mice. The expression of Prx-SO_3_ is highly increased in HCC of HBx Tg mice as compared to WT mice, as well as in dysplasia and hepatocellular adenoma (HCA) of HBx Tg mice, while pAkt and c-myc expressions were increased from earlier stage of tumorigenesis (Fig. 1), suggesting more severe oxidative stress in HCC.

ROS are important factors in the development of hepatic fibrosis. However, there is no evidence that the HBx-induced ROS expressions are directly involved in liver fibrosis. Necrosis was observed in only a few of our HBx Tg mice investigated (Tables 1, 2). Necrosis is followed by progressive fibrosis. As mentioned above, liver fibrosis in transgenic mice for HBV and HBx is induced by exposure to CCl4 [55–57]. CCl4 treatment is widely applied to produce liver fibrosis in normal mice and rats since this model most closely resembles clinical human liver cirrhosis among the different experimental models of liver fibrosis [76–78]. CCl_4_ treatment induces a profound elevation of ROS production and oxidative stress via the MAPK/NF-κB pathway [79]. Taken together, the above results indicate that the HBx-induced ROS is probably involved in promoting liver fibrosis caused by CCl4 treatment in normal mice.

## Conclusions

HBx Tg mice inducing HCC were generated by microinjecting the X-gene construct with the HBV-X gene under authentic promoter. Histological phenotypes associated with hepatic tumorigenesis were different, depending on the strain of HBx Tg mice, suggesting that genetic background affects phenotypes of HBx Tg mice.

Lipid accumulation and fatty changes were increased in the livers of HBx-Tg hybrid mice and the messenger RNA and protein levels of SREBP1 as well as PPARγ were also upregulated in HBx Tg mice. These results suggest that HBx protein induces hepatic steatosis in mice.

The role of HBx in regulating glucose metabolism was investigated in HBx-Tg B6 mice and HBx-Tg B6 mice lacking iNOS. Gluconeogenic genes were more significantly expressed in the livers of HBx-Tg B6 mice. This resulted in hyperglycemia, by increasing glucose production.

HBx correlates with the development of fibrosis. Convincing evidence is the induction of liver fibrosis in transgenic mice for HBV and HBx treated by carbon tetrachloride (CCl4). However, there is no evidence that the HBx is directly involved in liver fibrosis. The result indicates that HBx-induced ROS may be involved in promoting liver fibrosis induced by CCl4 treatment in normal mice.

ROS generation was remarkably increased in livers of HBx Tg young mice, suggesting that mitochondrial dysfunction induced by HBx expression increases the ROS generation, resulting in steatosis and inflammation.

Taken together, all the results indicate that HBx-induced ROS plays a role in inducing and promoting hepatic fatty change, glucose metabolism, and HCC.

## Data Availability

Not applicable.

## **Abbreviations**

CCl4: Carbon tetrachloride
CLD: Chronic liver disease
Cys): Cysteine
ECM: Extracellular matrix
H_2_O_2_: Hydrogen peroxide
HBV: Hepatitis B virus
HBx: HBV X
HBx-Tg B6: HBx C57BL/6 Tg mice
HBx-Tg hybrid: HBx hybrid C57BL/6 X CBA) Tg mice
HCC: Hepatocellular carcinoma
HCV: Hepatitis C virus
iNOS: Inducible nitric oxide synthase
LXRα: Liver X receptor α
Prxs: Peroxiredoxins
ROS: Reactive oxygen species
SREBP1: Sterol regulatory element binding protein 1
Tg: Transgenic
WT: Wild type
